# Optimization of Mycelia Selenium Polysaccharide Extraction from *Agrocybe cylindracea* SL-02 and Assessment of their Antioxidant and Anti-Ageing Activities

**DOI:** 10.1371/journal.pone.0160799

**Published:** 2016-08-17

**Authors:** Min Liu, Huijuan Jing, Jianjun Zhang, Gen Che, Meng Zhou, Zheng Gao, Shangshang Li, Zhenzhen Ren, Long Hao, Yu Liu, Le Jia

**Affiliations:** 1 Institute of Plant and Environment Protection, Beijing Academy of Agriculture and Forestry Sciences, Beijing Engineering Research Center for Edible Mushroom, Key Laboratory of Urban Agriculture (North), Ministry of Agriculture, Beijing, PR China; 2 College of Life Science, Shandong Agricultural University, Taian, PR China; 3 Shandong Academy of Agricultural Science, Ji’nan, PR China; 4 Quality and safety Monitoring Center of Animal Products, Ji’nan, PR China; University of Lancaster, UNITED KINGDOM

## Abstract

The aim of the present study was to optimize the purification of mycelia selenium polysaccharides (MSPS) from *Agrocybe cylindracea* SL-02 and characterize their *in vitro* antioxidant and *in vivo* anti-ageing activities. The Box-Behnken experimental design (BBD) was evaluated, which showed that the optimum conditions included an extraction temperature of 94.99°C, a pH of 9 and a precipitation temperature of 12°C, and the predicted yield was 11.036 ± 0.31%. The *in vitro* antioxidant assay demonstrated that MSPS had potential effects on scavenging and enhanced the reducing power of reactive oxygen species. The *in vivo* anti-ageing evaluation showed that MSPS significantly reduced the malonaldehyde (MDA) contents and total cholesterol (CHOL) levels, and remarkably improved the activities of superoxide dismutase (SOD), glutathione peroxidase (GSH-Px), and total antioxidant capacity (T-AOC) in mice in response to D-galactose-induced ageing. Furthermore, the characteristic analysis of MSPS indicated a selenium content of 1.76 ± 0.10 mg/g at a concentration of 6 μg/mL in liquid media and a monosaccharide composition of rhamnose, arabinose, mannose, glucose and galactose at a molar ratio of 29:3:1:18.8:2.7. These results suggest that MSPS might be suitable for functional foods and natural drugs on preventing the ageing progress induced by toxic chemicals.

## Introduction

Ageing, an inevitable process for all living organisms, damages the cell structure and promotes the disorder of physiological functions [1]. Several studies have reported that the ageing process involves many factors, including the accumulation of genomic mutations, toxic metabolites and free radicals; the hyposecretion of hormones; and the cross-linking of macromolecules under glycation [2]. However, the detailed mechanisms remain poorly understood [3]. One of the most popular theories for explaining the ageing process is the free radical theory [4], and an increasing number of studies have demonstrated that oxidative stress, followed by the overproduction of free radicals, plays a vital role in ageing [5]. As the balance of reactive oxygen species (ROS) production and antioxidant defence could determine the degree of ROS, oxidant intake and both dietary and synthetic antioxidants are beneficial to reduce the degree of ROS and confer protection against ageing. Nevertheless, synthetic antioxidants are restricted, reflecting the side effects of these compounds [6]. Hence, it is necessary to identify natural antioxidants with highly efficient and safe antioxidant properties to delay the ageing process. Therefore, the focus has recently changed to identifying harmless natural antioxidants from edible materials [7].

Currently, healthy diets have drawn increasing attention for their ability to retard the ageing process. Furthermore, the popularity of edible mushrooms has increased, as the polysaccharides of fungi possess immunoregulatory, antitumour, hypoglycaemic, antihyperlipidaemic and antioxidant activities [8]. *Agrocybe cylindracea*, one of the most precious edible and medicinal mushrooms industrially cultivated in China, contains high nutritional values and an attractive flavour. Recent studies have revealed that *A*. *cylindracea* has beneficial physiological activities, such as antitumour, anti-fungal, nerve tonic, lipid peroxidation inhibitory, anti-hypercholesterolaemia and anti-hyperlipidaemia activities [9]. However, there is a limited number of studies concerning the polysaccharide from *A*. *cylindracea* mycelia and its structure-function relationship.

Selenium, one of the essential trace elements in the human body, is involved in the synthesis of at least 30 antioxidant enzymes, particularly glutathione peroxidases (GSH-Px) [10]. Reflecting the low content of selenium in nature and common foods, selenium deficiency diseases are abundant worldwide. Therefore, there is a considerable demand for complementary and alternative medicines for the treatment of selenium deficiency. Interestingly, organic selenium generated through the biotransformation of mushrooms has received increasing attention as a result of its high bioavailability and low toxicity [11,12]. Because these mushrooms have higher bioaccumulation, the selenium content in mushroom-derived products can be improved through artificial cultivation on growth substrates with inorganic selenium. Although many reports have focused on the cultivation of fruit bodies [9,13,14], this strategy is time consuming and expensive. Although previous studies have reported that the bioaccumulation ability of fungi is species-specific and element-dependent [15], there are few studies concerning the combination of selenium with polysaccharides in mushrooms and the biological activities of selenium-polysaccharides.

In the present study, the conditions for purifying mycelia selenium polysaccharides (MSPS) from *A*. *cylindracea* were optimized using a Box-Behnken experimental design (BBD), a mathematical model that represents the relationship between the response and variables. The anti-ageing effects were analysed *in vitro* and *in vivo*, and the selenium accumulation rate, selenium content and monosaccharide composition were also processed.

## Experimental methods

### Chemicals and reagents

DEAE-52 cellulose, hydrogen peroxide (H_2_O_2_), ferrozine, 1,1-diphenyl-2-picrylhydrazyl (DPPH) and standard monosaccharides were purchased from Sigma Chemicals Company (St. Louis, USA). All other chemicals and reagents were analytical grade and purchased from local chemical suppliers in China.

### Fungal strains and culture conditions

The fungus strain *A*. *cylindracea* SL-02 was provided from Shandong Agricultural University and maintained on potato dextrose agar (PDA) slants (potato 200 g/L, dextrose 20 g/L, agar 20 g/L, MgSO_4_ 1 g/L and KH_2_PO_4_ 1.5 g/L). Liquid fermentation technology was used to produce *A*. *cylindracea* mycelia. After 14 days at 25°C, the *A*. *cylindrace* in Petri dishes was inoculated into 500 mL Erlenmeyer flasks containing 250 mL of medium and incubated on a rotary shaker at 120 rpm for 10 days at 25°C.

### Optimization of the Na_2_SeO_3_-concentration

Different concentrations of sodium selenite (Na_2_SeO_3_) (2, 4, 6 and 8 mg/L) were added to the substrates. After incubation, the mycelia of *A*. *cylindracea* were collected and weighed to obtain the best concentration. The mycelia were filtered and washed three times with deionized water, followed by constant drying at 50°C to measure the biomass (g/L). The mycelia (0.1 g) were nitrified through the addition of 2 mL of perchloric acid and 8 mL of nitric acid at room temperature for 12 h. The final 2 mL of nitrification liquor, determined through continuous heating, was mixed with 23 mL of double-distilled water for further flame atomic absorption spectrometry analysis (FAAS, nov AA^®^ 300, Analytik Jena AG, Jena, Germany).

### BBD optimization for MSPS extraction

Three parameters that significantly affect MSPS yields, including pH, extraction temperature and precipitation time, were selected for optimization through BBD. The test factors were coded according to the following equation:

$${\text{x}}_{i}=\frac{\left(X_{i}-X_{0}\right)}{\Delta X_{i}},\;i=1,\;2,\;3,\;\ldots \ldots ,\;k$$ (1)

where x_i_ and X_i_ represent the coded and actual values of independent variables, X_0_ is the actual value of the independent variable at the centre point and ΔX_i_ is the step change value. To correlate the response variable to the independent variable, the following quadratic polynomial equation was applied to fit the response variable to a quadratic model:

$$Y=\beta_{0}+\sum \beta_{i}x_{i}+\sum \beta_{ii}X_{i}{}^{2}+\sum \beta_{ij}x_{i}x_{j}$$ (2)

where Y is the predicted response value; β_0_, β_i_, β_ii_ and β_ij_ represent the intercept, linear, squared and interaction term, respectively; and x_i_ and x_j_ represent the coded levels of independent variables.

### Preparation of mycelia polysaccharides

The mycelia polysaccharides (MPS) and mycelia selenium polysaccharides (MSPS) were prepared as previously reported [16]. After washing three times with deionized water, the homogeneous mycelia powder was dried to constant weight at 50°C, pulverized using a mill and sieved through a 200-mesh screen. The MPS and MSPS were extracted in a water bath under the optimization conditions described above. Subsequently, the supernatant was centrifuged (3000 rpm, 10 min), concentrated and ethanol precipitated (1:4, v/v) at 4°C overnight. The polysaccharide precipitates were collected after centrifugation (3000 rpm, 10 min) and quantified using a phenol-sulfate method [17]. After lyophilization, the MPS and MSPS were collected for further analysis.

### Determination of monosaccharide composition

The monosaccharide composition was determined through gas chromatography (GC) (GC-2010, Shimadzu, Japan) on an Rtx-1 capillary column (30 m × 0.25 mm × 0.25 μm) according to Sheng et al. [18], with slight modifications. Briefly, the samples were hydrolysed with trifluoroacetic acid (TFA, 2 M, 110°C) for 4 h. After acetylation with hydroxylamine hydrochloride and pyridine, the hydrolysed supernatant (1 μL) was injected onto a column equipped with a flame ionization detector. Sugar identification was confirmed through comparison with standard monosaccharides of mannose, rhamnose, glucose, galactose, arabinose, D-ribose, xylose and inositol. The relative molar ratios were calculated using an area normalization method according to the chromatogram.

### Experiment of antioxidant properties *in vitro*

#### Scavenging assay of DPPH

The DPPH scavenging activity was determined according to Sun and Ho [19]. The reaction mixture contained DPPH-ethanol (2 mL, 0.1 mM) and sample (2 mL, 0–3000 mg/L). After shaking vigorously and incubating in the dark for 30 min, the absorbance was measured at 517 nm against a mixture of ethanol (2 mL) and distilled water (2 mL) as a blank.

The DPPH scavenging ability was expressed as:

$$Scavenging\;abilities\;(\%)=(1-\frac{A}{A_{0}})\times 100$$ (3)

Where A is the absorbance of the tested sample, and A_0_ is the absorbance of the blank.

#### Hydroxyl radical scavenging assay

The hydroxyl radical scavenging activity was determined according to Smirnoff and Cumbes [20], with some modifications. The H_2_O_2_ (1 mL, 8.8 mM) was added to initiate the reaction containing FeSO_4_ (1 mL, 9 mM), sodium salicylate-ethanol (1 mL, 9 mM) and sample (1 mL, 0–3000 mg/L) at 37°C for 0.5 h. After centrifugation (3000 rpm, 10 min), the absorbance was measured at 510 nm, with distilled water as a blank. For the control group, an equal amount of distilled water replaced the sample.

The hydroxyl radical scavenging activity was expressed as:

$$Scavenging\;rate\;(\%)=\frac{A_{0}-A_{1}}{A_{0}}\times 100$$ (4)

where A_0_ is the absorbance of the control group, and A_1_ is the absorbance of the sample.

#### Reducing power assay

The reducing power of MPS and MSPS was measured according to Oyaizu [21], with slight modifications. The reaction mixtures, containing 1 mL sample (0–3000 mg/L), 2.5 mL phosphate buffer (pH 6.6, 0.2 M) and 1 mL potassium ferricyanide (1%, w/v), were incubated at 50°C for 20 min and terminated after adding 2 mL trichloroacetic acid (10%, w/v). After centrifugation (1200 rpm, 10 min), the supernatant was collected and incubated with ferric trichloride (0.1%, 1.2 mL) for 15 min at room temperature. The absorbance was measured at 700 nm using distilled water as a blank.

### Anti-ageing *in vivo* experiments

Sixty Kunming mice (20 ± 2 g) were purchased from Taibang Biological Products Ltd. Co. (Taian, China), and the animal experiments were approved through the institutional animal care and use committee of Shandong Agricultural University in accordance with the Animals (Scientific Procedures) Act of 1986 (amended 2013). The mice were acclimatized for 7 d under controlled conditions (20–25°C, lights on 12 h daily) with diet and water *ad libitum*. All mice were randomly allocated into three control groups: normal (NC, n = 10), model (MC, n = 10) and test (n = 40) groups. The test group was further randomly and equally divided into low-dose (200 mg/kg) groups of MPS and MSPS (LM, LS) and high-dose (600 mg/kg) groups of MPS and MSPS (HM, HS). The normal and test groups were treated with 0.2 mL of distilled water through gastric gavage, and the test group was gavaged with 0.2 mL of different polysaccharides daily. Simultaneously, the normal group was administered physiological saline, and other groups were administered D-galactose (D-gal) (150 mg/kg) through intraperitoneal injection. After 20 days, the mice were fasted overnight and sacrificed through exsanguination under diethyl ether anaesthesia. The blood samples were obtained from the orbital sinus and centrifuged at 14000 rpm (4°C, 10 min) to afford the required serum. The livers were rapidly removed, weighed and immediately homogenized (1:9, w/v) in phosphate-buffered solutions (0.2 M, pH 7.4, 4°C). After centrifugation (5000 rpm, 4°C) for 20 min, the supernatants were collected for further biochemical analysis.

The GSH-Px activity, total antioxidant capacity (T-AOC), malonaldehyde (MDA) and total cholesterol (CHOL) contents in the liver and superoxide dismutase (SOD) activity in the serum were assayed using commercially available diagnostic kits (Nanjing Jiancheng Bioengineering Institute, Nanjing, China).

### Statistical analysis

All experiments were performed in triplicate, and the results are presented as the means ± standard deviation (SD). The results were analysed using one-way analysis of variance (ANOVA) with the IBM SPSS Statistical software package programme. *P* < 0.05 was considered statistically significant.

## Results and Discussion

### Determination of the Na_2_SeO_3_ concentration

**Fig 1:**
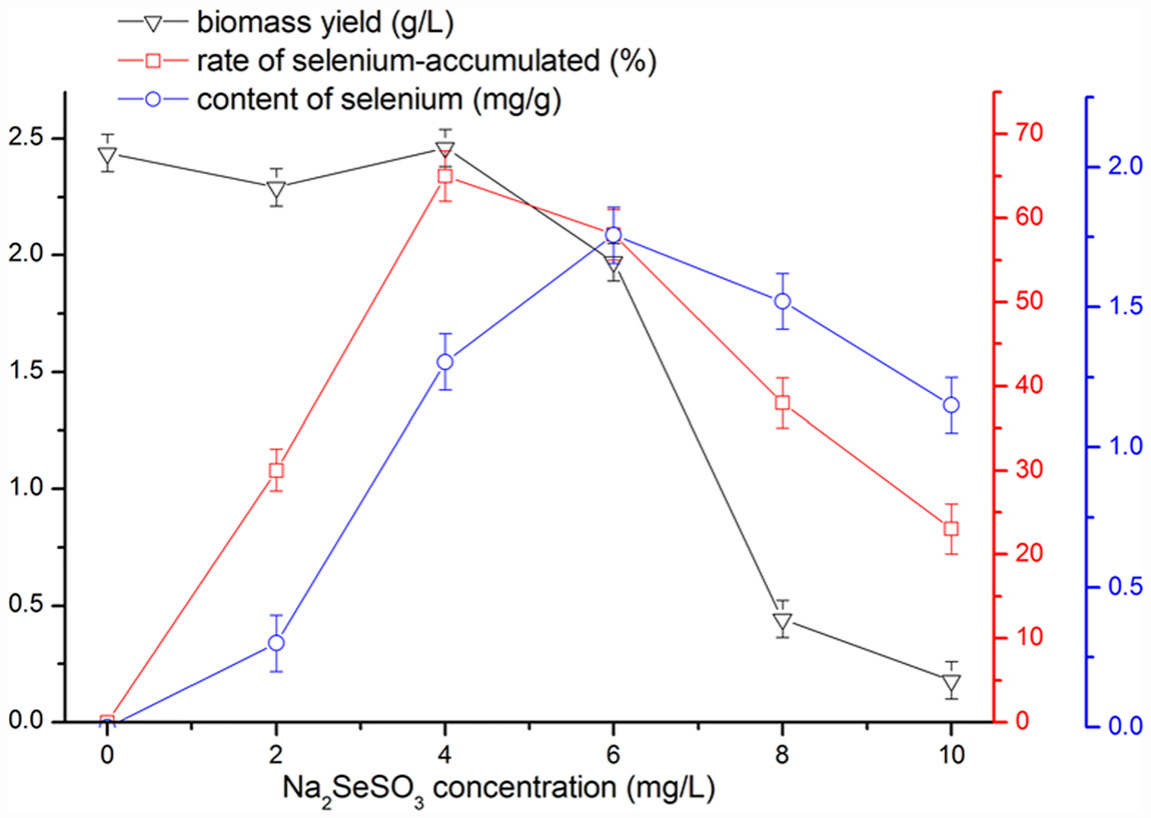
The biomass yield, selenium content and selenium accumulation rate of MSPS at different Na_2_SeO_3_ concentrations.

The Na_2_SeO_3_ concentration in the liquid medium was optimized and defined according to three indices: biomass yield, selenium accumulation rate and selenium content. As depicted in Fig 1, the three indices all presented increasing trends with increasing Na_2_SeO_3_ concentrations, peaking at 2.46 ± 0.08 g/L, 65.00 ± 3.00% and 1.76 ± 0.10 mg/g at the concentration of 4, 4 and 6 μg/mL, respectively. However, the trends were sharply reversed with increasing concentrations, potentially associated with mycotoxicity at high concentrations of elements, resulting in the inhibition of mycelial growth [22,23]. To determine the highest amount of selenium in the mycelia, the optimal Na_2_SeSO_3_ concentration of 6 μg/mL was used, and the biomass yield, selenium accumulation rate and selenium content were 1.97 ± 0.07 g/L, 58.00 ± 3.00%, and 1.76 ± 0.10 mg/g, respectively.

### BBD optimization of MSPS extraction

The BBD matrix and the experimental and predicted MSPS data are shown in Table 1, and the results of the ANOVA analysis are shown in Table 2. Using a multiple regression analysis, the polynomial model for the empirical relationship between the response and variables was expressed as

$$Y=8.86+0.61x_{1}+1.19x_{2}+0.40x_{3}+0.021x_{1}x_{2}+0.36x_{1}x_{3}-0.083x_{2}x_{3}-0.43x_{1}{}^{2}-0.43x_{2}{}^{2}+0.53x_{3}{}^{2}$$ (5)

where Y is the predicted response (Yields of MSPS, %), and x_1_, x_2_, and x_3_ represent the coded test variables for pH, extraction temperature (°C), and precipitation temperature (°C), respectively.

**Table 1:** Experimental and predicted values of MSPS based on central composite.

Run	X_1_ ^a^	X_2_ ^b^	X_3_ ^c^	MSPS yield (%)	
				Experimental	Predicted
1	-1 (5)	-1 (75)	0 (8)	6.10 ± 0.23	6.21
2	1 (9)	-1	0	7.35 ± 0.31	7.39
3	-1	1 (95)	0	8.60 ± 0.25	8.56
4	1	1	0	9.93 ± 0.32	9.82
5	-1	0 (85)	-1 (4)	8.39 ± 0.28	8.31
6	1	0	-1	8.81 ± 0.26	8.80
7	-1	0	1 (12)	8.38 ± 0.33	8.39
8	1	0	1	10.26 ± 0.35	10.34
9	0 (7)	-1	-1	7.31 ± 0.28	7.28
10	0	1	-1	9.71 ± 0.31	9.83
11	0	-1	1	8.37 ± 0.35	8.25
12	0	1	1	10.44 ± 0.34	10.48
13	0	0	0	8.98 ± 0.31	8.86
14	0	0	0	8.81 ± 0.28	8.86
15	0	0	0	8.89 ± 0.26	8.86
16	0	0	0	8.81 ± 0.24	8.86
17	0	0	0	8.81 ± 0.29	8.86

^a^: pH

^b^: Extraction temperature (°C)

^c^: Precipitation temperature (°C)

**Table 2:** ANOVA for the evaluation of the quadratic model.

Source	Coefficients	S.E.	Sum of squares	Mean square	*F*-value	*P*
Model	-	-	18.92	2.1	157.69	<0.0001
Intercept	8.86	0.052	-	-	-	-
x_1_	0.61	0.041	2.98	2.98	223.58	<0.0001
x_2_	1.19	0.041	11.4	11.4	855.36	<0.0001
x_3_	0.4	0.041	1.3	1.3	97.69	<0.0001
x_1_x_2_	0.021	0.058	1.71E-03	1.71E-03	0.13	0.7306
x_1_x_3_	0.36	0.058	0.53	0.53	39.79	0.0004
x_2_x_3_	-0.083	0.058	0.027	0.027	2.06	0.1948
x_1_^2^	-0.43	0.056	0.79	0.79	59.41	0.0001
x_2_^2^	-0.43	0.056	0.79	0.79	59.41	0.0001
x_3_^2^	0.53	0.056	1.2	1.2	90.29	<0.0001
Lack-of-fit			0.072	0.024	4.19	0.1001
Residual			0.094	0.013		
Pure error			0.023			
Cor total			18.99			
Mean	8.7					
c.v.%	1.33					
Adeq Precision	47.837					
R-squared	0.995					
Adj R-squared	0.9886					
Pred R-squared	0.9378					

The results of the ANOVA, a statistical technique used to subdivide the total variations into component parts associated with specific sources of variation to examine hypotheses on the parameters, are shown in Table 2. The linear term regression coefficients (x_1_, x_2_, and x_3_), quadratic coefficients (x_1_^2^, x_2_^2^, and x_3_^2^) and interaction coefficient (x_1_x_3_ and x_2_x_3_) were significant at the 1% level, indicating that pH, extraction temperature and precipitation temperature were all significantly correlated with the MSPS yield. The large model *F*-value (157.69) and the low Lack-of-Fit *F*-value (4.19) suggest that most of the variation in MSPS yield reflects this regression equation, demonstrating that the developed quadratic models were significant to predict the MSPS yield.

In addition, the variance analysis, including the mean, coefficient of variation (C.V., %), Adeq precision, R-squared, Adj R-squared and pred R-squared values were calculated to assess the adequacy and accuracy of the developed models. The R-squared value showed the proportion of the total variation in the response predicted using the model. A high R-squared value of 0.9950 in the present study ensured satisfactory fitness to represent the actual relationship between the responses and the variables. The Adj R-squared and pred R-squared values represented the amount of variation around the mean explained by the model, adjusting for the number of terms in the model. The current Adj R-squared and pred R-squared values indicated that the selected terms significantly contributed to the model, and almost 93.78% of the variability in predicting new observations in the design space could be explained in this model. Furthermore, as a significant method to measure the unexplained or residual variability of the data as a percentage of the mean of the response variable, a low C.V.% value of 1.33 in the present study indicated a high degree of precision and a good deal of experimental values reliability [24,25]. In conclusion, the model equation was appropriate to predict the MSPS yield under any combination of values.

**Fig 2:**
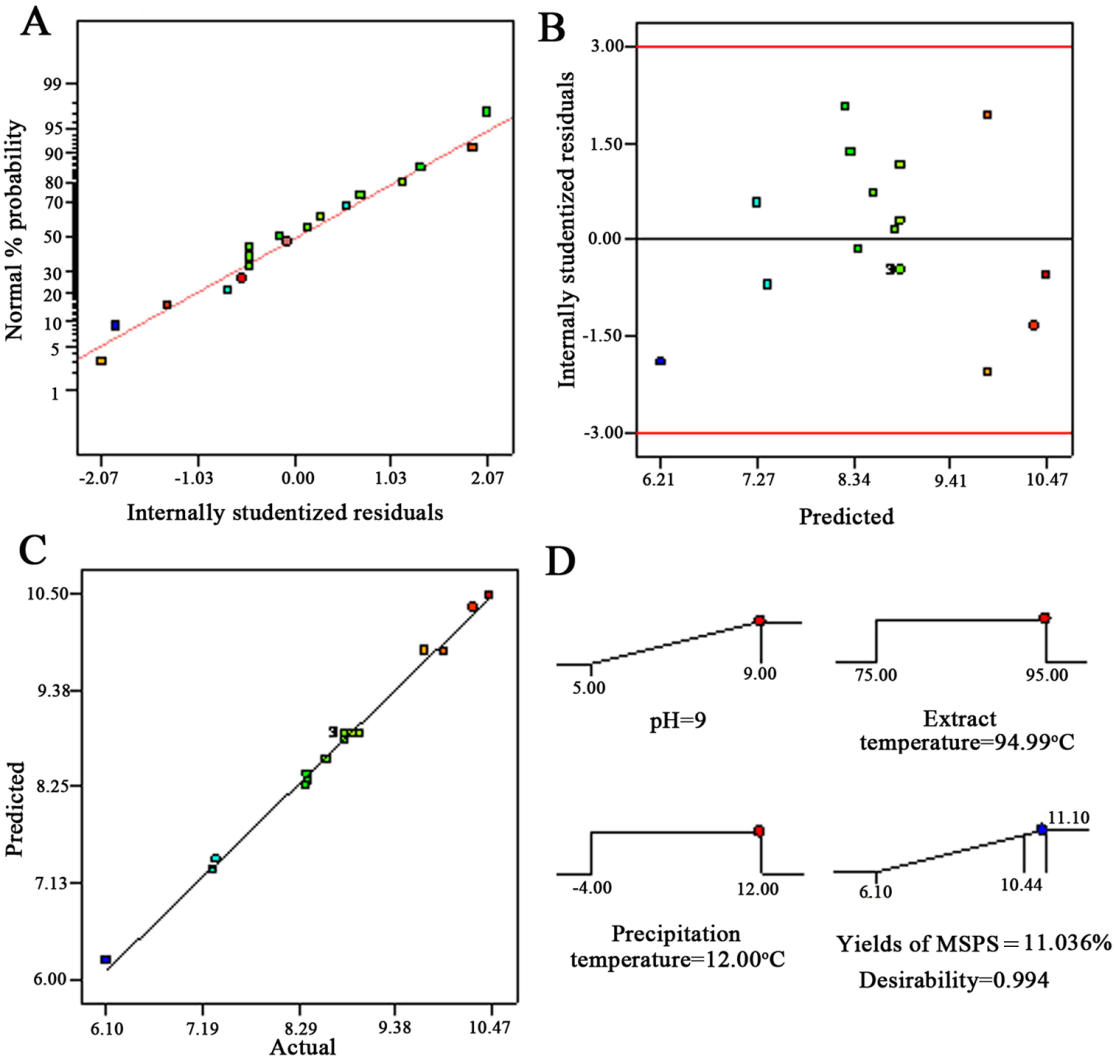
Diagnostic plots for the Box-Behnken model adequacy. (A) Normal plot of residuals, (B) plot of internally studentized residuals vs. predicted response, (C) plot of internally studentized residuals vs. actual and (D) desirability ramp plot for optimization.

The adequacy of the model was also evaluated after inspecting the diagnostic plots of the residuals, residuals vs. predicted and predicted vs. actual values (Fig 2). The data were analysed to assess the normality of the residuals to the determination coefficient. Hifney et al. [25] showed that residuals following a normal distribution should form a straight line when the values are fitness under the theorized model. As shown in Fig 2(A), these valued formed a straight line, and the normal plot of residuals for MSPS yields was normally distributed, indicating no deviation of the variance. No clear patterns were observed in the residuals vs. predicted plot, validating the initial assumption of constant variance (Fig 2(B)). In addition, the predicted vs. actual values plots also showed excellent agreement (Fig 2(C)). Hence, the adequacy of the present model was well established.

By solving Eq 5, the optimal conditions for obtaining the maximum MSPS yield (11.036 ± 0.31%) were pH of 9, 94.99°C extraction temperature and 12°C precipitation temperature, and the ramp desirability figure showed 0.994 desirability, a value close to 1, indicating that this model could make significant contributions to an economically advantageous factor for extracting MSPS.

### Monosaccharide composition

**Fig 3:**
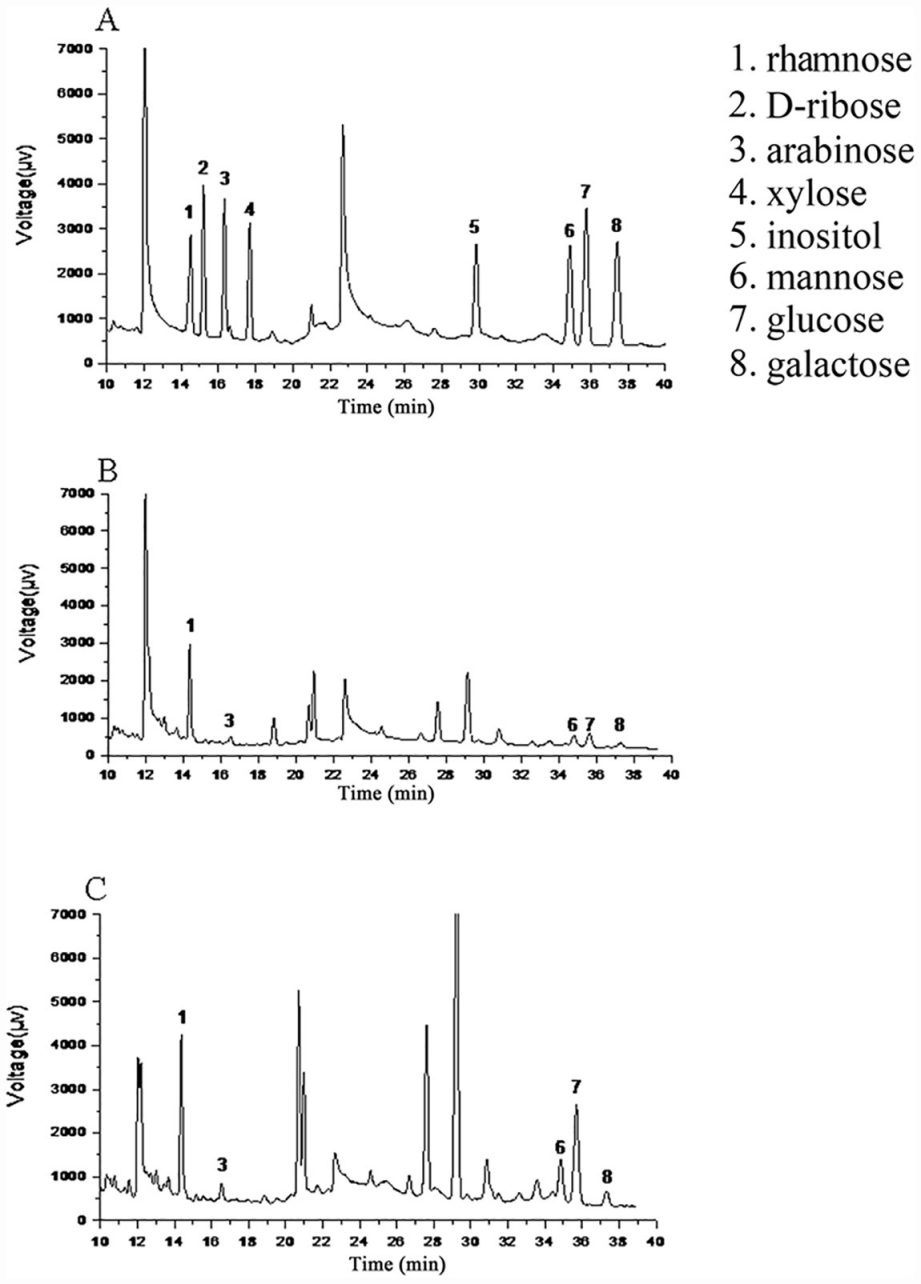
HPLC chromatograms of polysaccharides. (A) Standard, (B) MSPS, and (C) MPS.

The HPLC chromatograms of MSPS and MPS are shown in Fig 3. Glucose was the major component of all polysaccharides. MSPS comprised rhamnose, arabinose, mannose, glucose and galactose at a molar ratio of 29:3:1:18.8:2.7, while MPS comprised rhamnose, arabinose, mannose and glucose at a molar ratio of 29.2:1.8:3:4. These results showed that the major monosaccharide component in MSPS and MPS was glucose, and galactose was present only in MSPS, indicating that both glucose and galactose could maintain the antioxidant activities of polysaccharides. Capek et al. reported similar results, showing that galactose had superior abilities of enhancing antioxidant activities [26].

### Antioxidant properties *in vitro*

**Fig 4:**
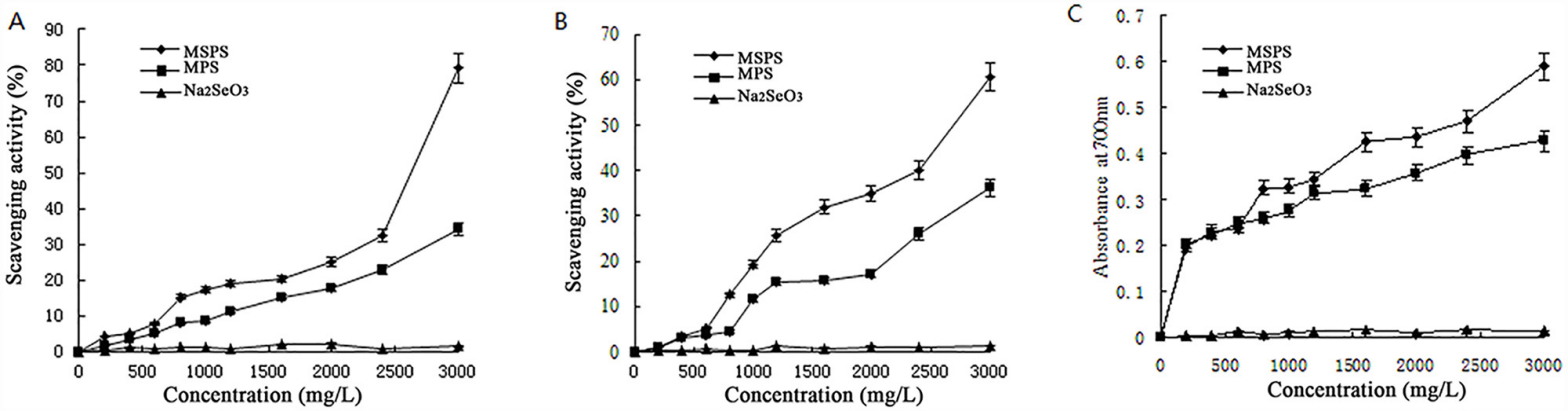
Antioxidant capacities of the polysaccharides *in vitro*. (A) DPPH radical-scavenging activity, (B) hydroxyl radical-scavenging activity and (C) reducing power.

Previous studies have reported that microelements potentially enhance the abilities of biomacromolecules, such as polysaccharides for scavenging radicals [27] and proteins for antioxidation [10]. Because free radicals are the major cause of ageing, antioxidant scavenging could be an indicator of anti-ageing activities. Selenium is an essential dietary trace element that plays an important role in a number of ageing processes, and a recent widely publicized study showed that selenium supplements delay senescence [28,29]. The aim of the present study was to evaluate the absorbance of selenium by fungi and the anti-ageing properties of selenium-polysaccharide. As depicted in Fig 4, three typical indices were selected to determine the antioxidant abilities of MSPS and MPS using Na_2_SeO_3_ as the control material.

DPPH, a relatively stable radical widely used to investigate the scavenging activity of some antioxidants, can accept an electron or hydrogen atom to become a stable diamagnetic molecule [30]. The radical form of DPPH could be scavenged by an antioxidant into a non-radical DPPH form, thereby reducing the absorbance [31]. As shown in Fig 4(A), the scavenging ability of MSPS reached 79.13 ± 0.23% at 3000 mg/L, which was 131.6% higher than the MPS concentration. The effect of MSPS on DPPH scavenging was better than that of the hot water *A*. *cylindracea* extracts [32].

Hydroxyl radical (HO·), a natural by-product, can attack biological molecules, such as lipids, proteins, enzymes, DNA and RNA, leading to cell or tissue injury associated with degenerative diseases [33,34]. As illustrated in Fig 4(B), the scavenging ability of MSPS reached 60.54% (67.3% higher than that of MPS), indicating that MSPS has potential antioxidant abilities *in vitro*.

Reducing agents might serve as significant indicators of potential antioxidant activity [35]. As shown in Fig 4(C), the reducing power of MPS and MSPS exhibited a dose-dependent effect. The reducing power of MSPS reached 0.61 ± 0.09 at 3000 mg/L, which was 69.4% higher than that of MPS, indicating that MSPS had better potential antioxidant properties than MPS *in vitro*.

In addition, as an inorganic compound, Na_2_SeO_3_ showed scarce antioxidant abilities at any concentration, demonstrating the prominent roles of fungi in the biotransformation of elements.

### Anti-ageing abilities

As selenium has a narrow range between dietary deficiency (< 40 μg/day) and toxic levels (> 400 μg/day), the current recommended daily dietary intake of selenium for humans is 57 μg/d [36]. Although the demand for selenium is low, the content of this element in food is typically insufficient, and the chemical form of selenium is limited. For the mice used in the present study, the daily dietary intake of polysaccharides was 600 and 200 mg/kg.

D-gal has been widely used to induce age-related damage in rodents, based on the production of free radicals and the acceleration of senescence [37]. The natural ageing process in humans has been associated with free radicals, which severely damage adjacent biomolecules, such as proteins, DNA, fatty acids and nucleic acids. The pathogenesis of ageing through D-gal is oxidative damage. Antioxidants might play an important role in preventing free radical damage associated with ageing, interfering directly in the generation or scavenging of radicals. Previous studies have indicated that mushroom polysaccharides, as antioxidants, can limit the degree of ROS [38]. Furthermore, the co-production of selenium and other antioxidants show antioxidant effects on radicals and lipid peroxidation production [39]. Therefore, we examined the co-effect of selenium and mycelia polysaccharides on anti-ageing.

**Fig 5:**
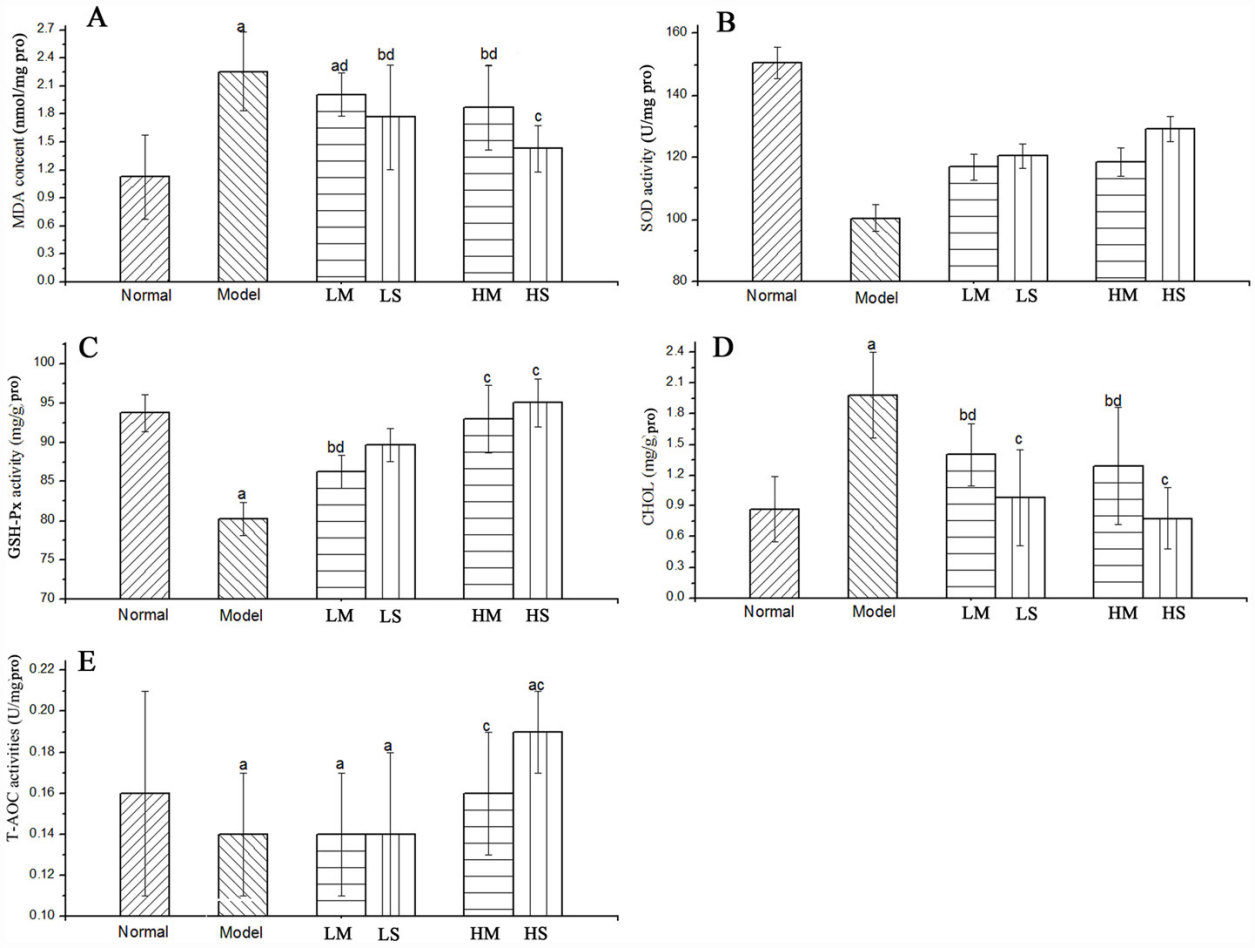
Hepatic parameters of (A) MDA content, (C) GSH-Px activity, (D) CHOL level and (E) T-AOC activity and serum parameters of (B) SOD activity ^a^ p < 0.01 compared with the normal control group.

As shown in Fig 5(A), compared with the model control group, the MDA contents in LS, HS, LM and HM in the liver were reduced 21.7%, 36.7%, 11.1% and 17.3% in dose-dependent patterns at the tested concentrations, respectively. The SOD activities in LS, HS, LM and HM were increased 20.0%, 28.6%, 16.5% and 18.1%, respectively, compared with the model group. The GSH-Px activities of HS were significantly increased 5.89% compared with HM, indicating that MSPS had higher activities than MPS. The liver cholesterol levels (Fig 5(D)) in LS, HS, LM and HM were 50.5%, 60.6%, 29.3% and 34.8% lower, respectively, than in the model group. As shown in Fig 5(E), neither LM nor LS showed differences from the model group in the T-AOC index; however, the levels of T-AOC in HM and HS were higher than in the model control group and increased in a dose-dependent manner. HS and HM were 35.7% and 14.3% higher, respectively, than the model group. HS was 18.8% higher than the normal group, showing that MSPS has stronger antioxidant activity.

The overproduction of radicals induced through D-gal increased the lipid peroxide levels and decreased enzyme activity, preventing lipid peroxidation in the tissues. For the analysis of lipid peroxide, MDA, an indicator of oxidative stress, is the main decomposition product of peroxides derived from polyunsaturated fatty acids, which determines the lipid peroxidation levels [40]. A significant increase in the MDA content (Fig 5(A)) was observed in the D-gal model control group compared with the normal control group, indicating that high-dose MSPS could relieve the mice undergoing D-gal treatment. For the analysis of enzymatic reactions, SOD is the first and most important antioxidant enzyme defence system against oxidative stress, converting the superoxide radical to H_2_O_2_ through GSH-Px [41,42]. In addition, SOD catalyses the dismutation of superoxide radicals into oxygen and hydrogen peroxide, thereby participating with other antioxidant enzymes in the enzymatic defence against oxidative injury [43]. GSH-Px, with selenium as an essential factor, is located in the cytosol of most cells and is responsible for the reduction of hydro and organic peroxides during senescence. Selenium plays an antioxidant role with GSH-Px, as this co-factor reduces hydrogen peroxides, lipids and phospholipid hydroperoxides [44]. Accordingly, T-AOC represents an original enzymatic and non-enzymatic antioxidant in mice. Compared with the normal group, a significant decrease of T-AOC was observed in the model group, indicating that that the model was successfully established. Cholesterol is a waxy, fat-like substance naturally occurring in all parts of the body; when in excess, cholesterol can cause heart disease. In summary, these observations indicate that MSPS has anti-ageing activity and could significantly counteract increased oxidative stress through the promotion of enzymatic and non-enzymatic antioxidant activities, thereby reducing levels of lipid peroxides.

## Conclusion

The Na_2_SeO_3_ concentration in the liquid medium used to produce MSPS of *Agrocybe cylindracea* SL-02 was determined, and BBD was a successful tool for optimizing the extraction MSPS. In addition, MSPS exhibited anti-ageing activities *in vivo* and antioxidant activities *in vitro*. In summary, the selenium-enriched mycelia of *A*. *cylindracea* represent a novel dietary source of bioavailable supplemental selenium.

## Supporting Information

S1 ARRIVE Checklist (DOCX)

## Abbreviations

ANOVA: Analysis of variance
BBD: Box-Behnken experimental design
CHOL: Total cholesterol
D-gal: D-galactose
DPPH: 1,1-diphenyl-2-picrylhydrazyl
GC: Gas chromatography
GSH-Px: Glutathione peroxidase
HO: Hydroxyl radical
H_2_O_2_: Hydrogen peroxide
MDA: Malonaldehyde
MPS: Mycelia polysaccharide
MSPS: Mycelia selenium polysaccharides
MC: Model control group
NC: Normal control group
Na_2_SeO_3_: Sodium selenite
PDA: Potato dextrose agar
ROS: Reactive oxygen species
SD: Standard deviation
SOD: Superoxide dismutases
T-AOC: Total antioxidant capacity
