# Supplementary material for: Optimization of Mycelia Selenium Polysaccharide Extraction from Agrocybe cylindracea SL-02 and Assessment of their Antioxidant and Anti-Ageing Activities
Source: PLoS One. 2016 Aug 17;11(8):e0160799. doi: 10.1371/journal.pone.0160799 (PMC4988633; doi:10.1371/journal.pone.0160799)

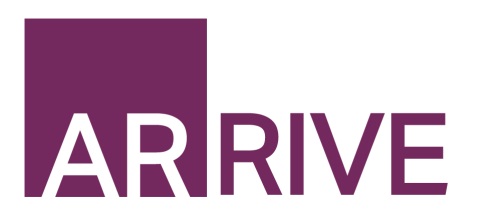


The ARRIVE Guidelines Checklist

Animal Research: Reporting In Vivo Experiments

Carol Kilkenny^1^, William J Browne^2^, Innes C Cuthill^3^, Michael Emerson^4^ and Douglas G Altman^5^

*^1^The National Centre for the Replacement, Refinement and Reduction of Animals in Research, London, UK, ^2^School of Veterinary Science, University of Bristol, Bristol, UK, ^3^School of Biological Sciences, University of Bristol, Bristol, UK, ^4^National Heart and Lung Institute, Imperial College London, UK, ^5^Centre for Statistics in Medicine, University of Oxford, Oxford, UK.*

|  | ITEM | RECOMMENDATION | Section/ Paragraph |
| --- | --- | --- | --- |
| 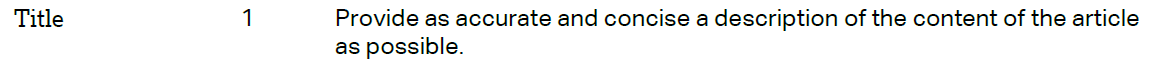 | | | **Title** |
| 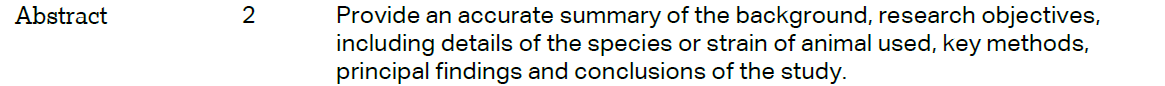 | | | **Abstract** |
| INTRODUCTION | | |  |
| 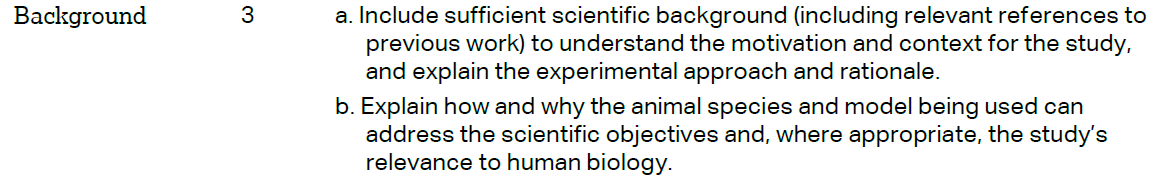 | | | **Paragraphs 1-3**  **N/A** |
| 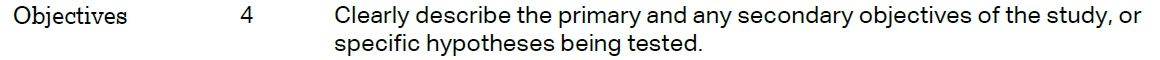 | | | **Paragraphs 4** |
| METHODS | | |  |
| 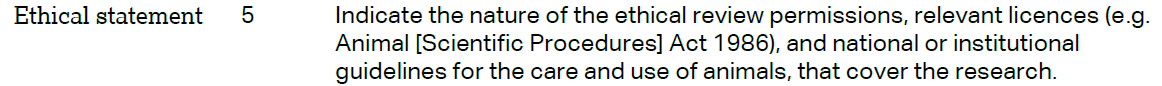 | | | **Section 2.8** |
| 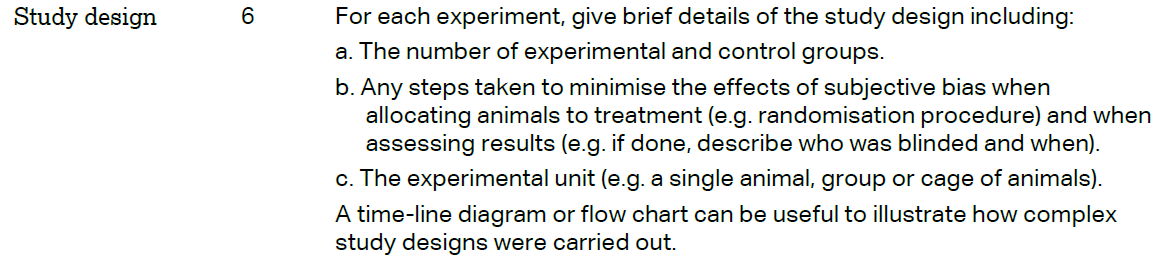 | | | **Section 2.8** |
| 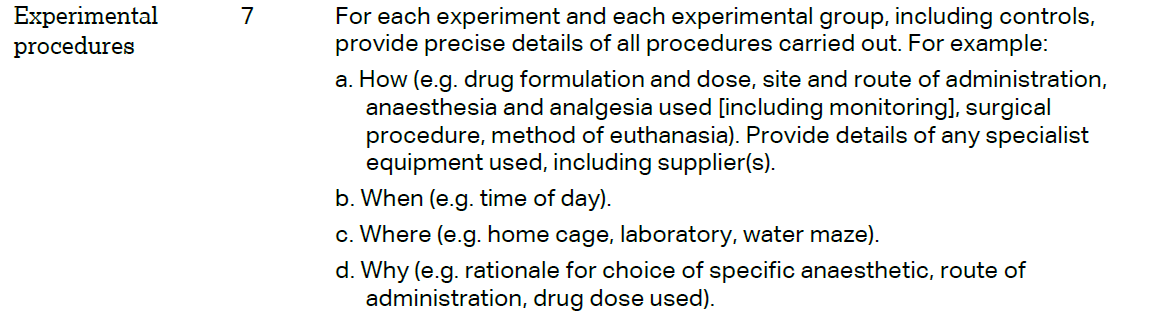 | | | **Section 2.8** |
| 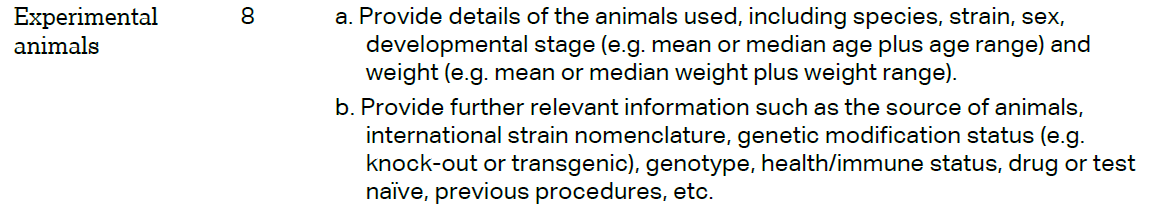 | | | **Section 2.8** |

The ARRIVE guidelines. Originally published in *PLoS Biology*, June 2010^1^

| 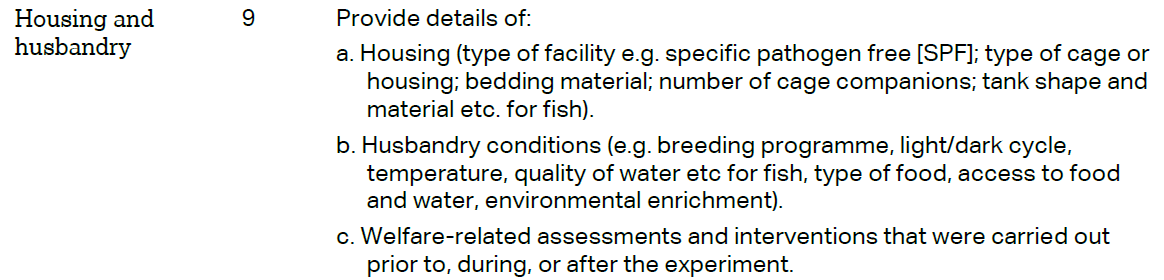 | **Section 2.8** | |
| --- | --- | --- |
| 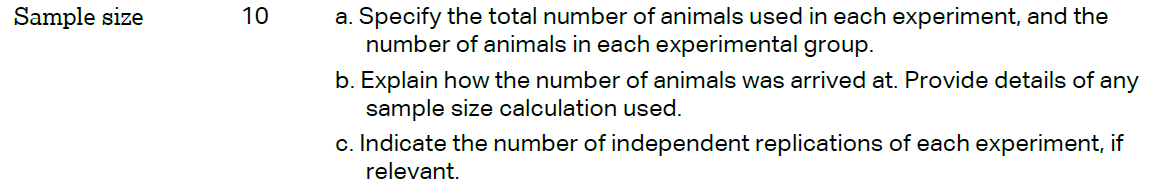 | **Section 2.8** | |
| 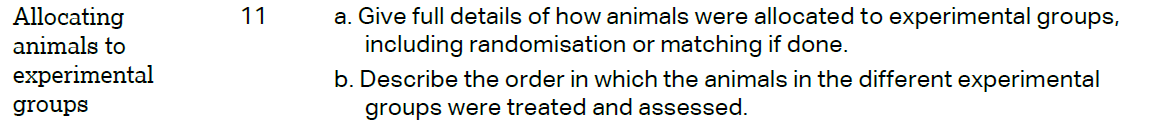 | **Section 2.8** | |
| 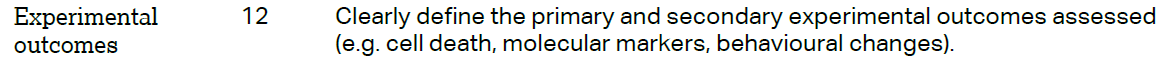 | **Section 2.8** | |
| 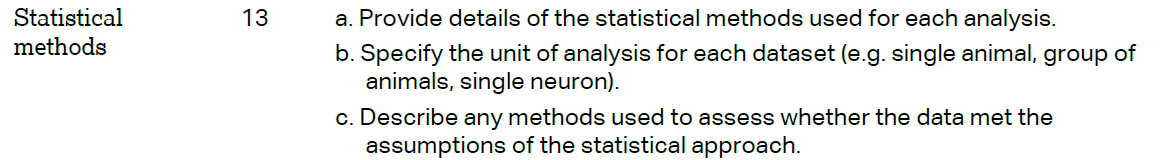 | **Section 2.8 and Section 2.9** | |
| RESULTS |  | |
| 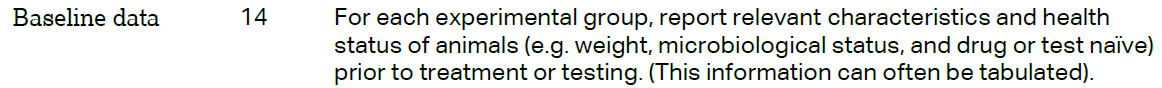 | **Section 2.8** | |
| 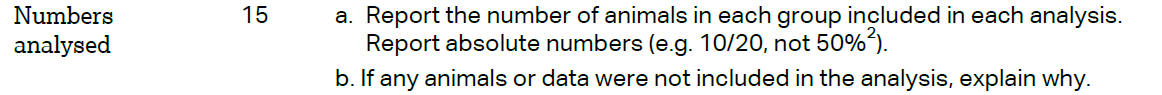 | **Section 2.8**  **N/A** | |
| 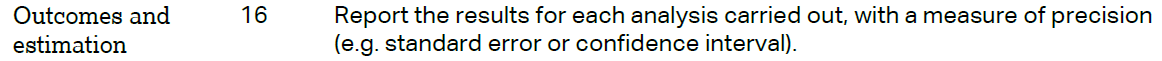 | **Section 2.8** | |
| 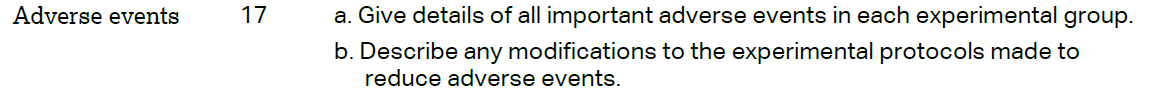 | **N/A** | |
| DISCUSSION |  | |
| 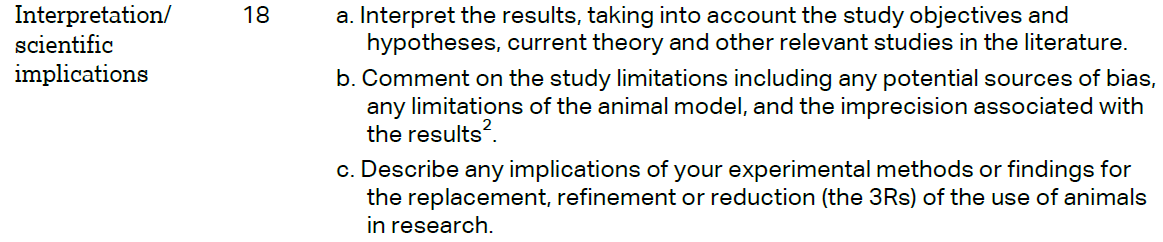 | **Paragraphs 1**  **N/A**  **N/A** | |
| 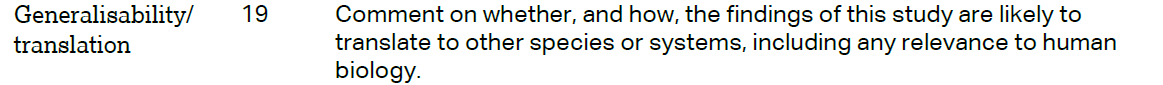 | **Section 3.5** | |
| 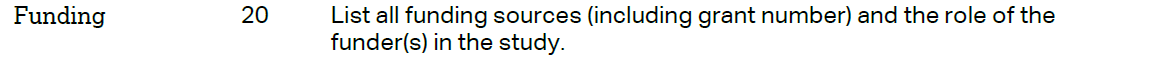 | **Acknowledgement** |  |


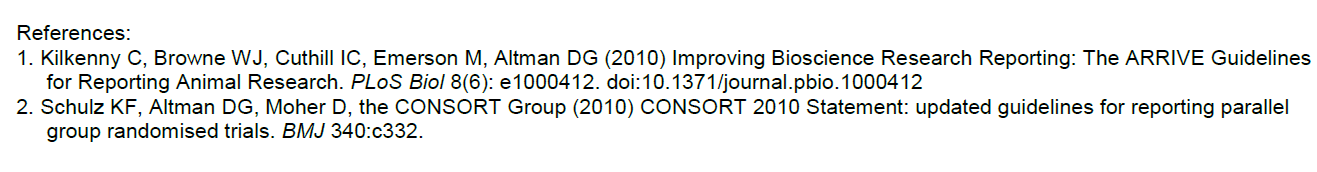

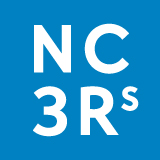

Supplement: S1 ARRIVE Checklist — (DOCX) [file pone.0160799.s001.docx]
